# Prevalence of low-level viremia and related influencing factors among people living with HIV in China: a systematic review and meta-analysis

**DOI:** 10.3389/fpubh.2025.1661253

**Published:** 2025-10-02

**Authors:** Xinxin Zhang, Qianlei Xu, Chen Li, Yue Zhang, Yantao Jin, Pengyu Li, Huijun Guo

**Affiliations:** ^1^AIDS Clinical Research Center, The First Affiliated Hospital of Henan University of Chinese Medicine, Zhengzhou, China; ^2^First Clinical Medical College, Henan University of Chinese Medicine, Zhengzhou, China

**Keywords:** HIV, AIDS, low-level viremia, prevalence, meta-analysis, China

## Abstract

**Introduction:**

The promotion and application of ART have significantly reduced the mortality rate of AIDS patients in China, but the problem of LLV that follows cannot be ignored. LLV is closely associated with viral failure and increased all-cause mortality. Conducting a systematic study on the prevalence of LLV among PLWH in China and its influencing factors has significant clinical implications for the comprehensive management, effective prevention, and intervention of LLV in the future.

**Methods:**

Studies were included if research subjects were PLWH in China, with a duration of ART treatment of ≥6 months, the research types were cohort studies, case–control studies, and cross-sectional studies, the primary outcome indicator was LLV occurrence, as well as research findings were disseminated in Chinese or English. The systematic review was conducted using the R studio software.

**Results:**

Our review included a total of 20 original studies, covering 225,687 HIV/AIDS patients, among which 45,265 were LLV. Our study showed that the overall prevalence rate of LLV among PLWH in China was 10.6% (95% CI: 6.8% ~ 16.1%). Further subgroup analysis revealed that Blip was higher than pLLV, the prevalence of LLV was the highest in the VL range of 50 ~ 199 cp/mL; there were no significant differences in the prevalence of LLV among different regions, type of study and ART regimens. The analysis of factors influencing LLV showed that poor ART adherence, baseline VL > 10^5^ cp/mL, changing the treatment schemes, baseline CD4 < 350 cells/μL, PI-based regimen at initiation, age ≥50 years, and age of ART initiation ≥50 years were risk factors for LLV among PLWH, while homosexual transmission was a protective factor for LLV (*p* < 0.001).

**Conclusion:**

The prevalence of LLV among PLWH was moderate to low in China. Special attention should be paid to older population with PLWH who had poor ART adherence, high baseline VL, had changed the treatment schemes, had a low baseline CD4 level, had an PI-based regimen at initiation, started ART relatively late, and needed enhanced compliance management and drug resistance testing, as well as increased VL testing frequency to avoid adverse outcomes.

**Systematic review registration:**

https://www.crd.york.ac.uk/PROSPERO/view/CRD420251047912, identifier (CRD420251047912).

## Background

By the end of 2023, an estimated 39.9 million individuals were living with HIV globally, of whom 30.7 million were receiving antiretroviral therapy (ART) (1). In China, 31 provinces reported 1,355,017 current people living with HIV(PLWH) by the end of 2024 (2). The ART coverage rate in China increased from 74.04% in 2016 to 92.9% in 2020, maintaining stability thereafter. ART effectively suppresses viral replication, reducing HIV viral load (VL) to undetectable levels and realizing the “U=U” principle (Undetectable = Uninfectious) (3). However, emerging evidence shows that persistent viral suppression is not achieved in all patients, leading to low level viraemia (LLV).

The US Department of Health and Human Services defines LLV as VL < 200 cp/mL (4); the Chinese AIDS Diagnosis and Treatment Guidelines (2024 Edition) specify VL of 50 ~ 200 cp/mL (5); while the World Health Organization and China’s National Free Antiviral Drug Treatment Manual (5th Edition) define it as VL between 50 and 1,000 cp/mL (6, 7). Evidence indicates that LLV among PLWH retains the potential for HIV transmission (8). LLV promotes drug resistance mutations, increasing the risk of virological failure (VF), and exacerbates immune activation and inflammatory responses, thereby elevating the risk of AIDS defining and non AIDS defining diseases (9–11). Compared to virologically suppressed PLWH, individuals with LLV exhibit a significantly elevated risk of all-cause mortality, warranting heightened attention to their disease management (12, 13).

China has made substantial progress in HIV/AIDS prevention and control, with both treatment coverage and viral suppression rates exceeding 90%, keeping the national epidemic at a low-prevalence level (14). However, the prevalence of LLV has risen gradually in recent years, surpassing 30% in certain provinces (15). This not only poses a significant threat to the survival of PLWH but also emerged as a critical gap hindering further improvements in the quality of China’s HIV/AIDS prevention and control efforts. Evidence serves as the cornerstone of clinical decision making, with high quality evidence providing invaluable references for researchers and clinicians (16). An integrated analysis of the prevalence and influencing factors of LLV among PLWH in China would facilitate the optimization of national HIV/AIDS prevention and control strategies and the standardized management of LLV. Accordingly, this study aims to synthesize existing clinical evidence via meta-analysis to characterize the current landscape of LLV among PLWH. The ultimate goal is to provide evidence-based support for the development of effective LLV intervention and prevention measures, as well as the formulation of personalized treatment regimens.

## Materials and methods

### Design and registration

This systematic review and meta-analysis were designed, conducted, and reported based on the Preferred Reporting Items for Systematic Reviews and Meta-Analyses (PRISMA) (17). This study was registered on PROSPERO (registration number: CRD420251047912).

### Search strategy

A systematic review of literature was performed using eight databases: (1) PubMed; (2) EMBASE; (3) Cochrane Library; (4) Web of Science; (5) China National Knowledge Infrastructure (CNKI); (6) Wanfang; (7) Weipu database (VIP), and (8) China Biology Medicine disk (CBM). Search through various databases for papers published from their establishment until April 22, 2025. Keywords and MeSH terms synonymous with “HIV,” “AIDS,” “Human Immunodeficiency Viruses,” “Acquired Immunodeficiency Syndrome,” “low level viraemia,” “hypoviremia,” “low level viremia.” In addition, reference lists from previous related reviews were screened to ensure a comprehensive search. Details of the search strategies are provided in Supplementary Table S1.

### Inclusion and exclusion criteria

Inclusion criteria: (1) The research subjects were PLWH in China, with a duration of ART treatment of ≥6 months; (2) The research types were cohort studies, case–control studies, and cross-sectional studies; (3) The primary outcome indicators were LLV occurrences, and the secondary outcome indicators were the influencing factors of LLV; (4) Research findings disseminated in either Chinese or English.

Exclusion criteria: (1) Review articles, scientific achievements, news reports, etc.; (2) Studies for which the full text cannot be obtained or the data cannot be extracted; (3) Republished literature; (4) Studies with a sample size of less than 300.

The specific definitions are as follows: (1) LLV, defined as the occurrence of at least one VL measurement of 50 ~ 1,000 cp/mL after virologic suppression is achieved or ART treatment of ≥6 months; (2) persistent LLV (pLLV), defined as two or more consecutive VL of 50 ~ 1,000 cp/mL after virologic suppression is achieved or ART treatment of ≥6 months, and Blip defined as only once or multiple times at intervals.

### Data extraction

Two investigators independently extracted data using Excel spreadsheets, collecting information on first author, publication year, study design, geographic region, total sample size, LLV cases, LLV incidence, and multivariate analysis factors. In case of any disagreement, a third researcher would make the final decision.

### Quality assessment

For cohort and case–control studies, literature quality was assessed using the Cochrane recommended Newcastle-Ottawa Scale (NOS), a 9-point tool where 0–3 points indicated low quality, 4–6 moderate quality, and 7–9 high quality. Cross-sectional studies were evaluated with the US Health Care and Research Institutions Quality Assessment Scale (11-point total), classifying 0–3 points as low, 4–7 as moderate, and 8–11 as high quality.

### Statistical analysis

This meta analysis was performed using R Studio software. The prevalence of LLV was calculated for each included study using the total number of PLWH and the number of PLWH with LLV. The variation among the studies was evaluated through the application of Cochran’s Q statistic and the I-square statistics (I^2^). If *I*^2^ ≤ 50% and *p* ≥ 0.10, no significant heterogeneity was indicated among the included studies, and a common effect model was used for the meta analysis; if *I*^2^ > 50% and *p* < 0.10, substantial heterogeneity was suggested, and a random effects model was applied instead. Sensitivity and subgroup analyses were conducted to further investigate heterogeneity and validate the robustness of the study findings. The subgroup analyses examined the prevalence of LLV by frequency of LLV, VL level, geographic regions, type of study and treatment regimens. A funnel plot, Egger’s test and Begg’s test were used to analyze the publication bias in the studies included. The trim-and-fill method was used to identify and correct potential publication bias. For influencing factor analysis, odds ratios (OR) with 95% confidence intervals (CI) were used to pool effect sizes. All statistical tests were two-sided, with statistical significance set at *p* < 0.05.

## Results

### Literature screening process and results

**Figure 1:**
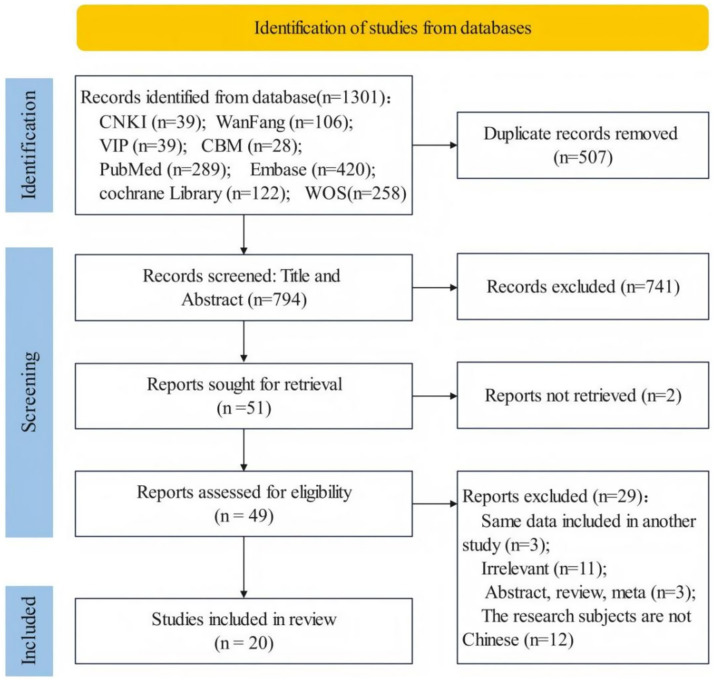
Literature screening flowchart.

Figure 1 shows the steps involved in searching for and screening literature. A total of 1,301 studies were retrieved by the database searches. After removing duplicates and screening titles, abstracts and full text, 20 studies (12 Chinese and 8 English studies) were included (15, 18–36) (Figure 1).

### Basic characteristics of the included studies

Table 1 summarizes the study’s baseline characteristics. A total of 20 literatures were finally included, involving 225,687 PLWH. The sample size ranged from 402 to 93,944 cases, predominantly male, among which 45,265 were LLV patients. The prevalence rate of LLV among PLWH ranges from 1.3 to 38.7%. The studies were conducted across 12 Chinese provinces, municipalities, and autonomous regions, with higher representation from Beijing (*n* = 4), Guangdong (*n* = 2), Henan (*n* = 2), Liaoning (*n* = 2), and Taiwan (*n* = 2). Geographic distribution showed a southern region predominance (*n* = 12). Publication years spanned 2019–2024, and the number of published literatures was the largest in 2022 (*n* = 9), followed by 2023 (*n* = 4) and 2024 (*n* = 4). The research types were dominated by retrospective cohort studies (*n* = 17).

**Table 1:** Characteristics of the included studies.

References	Year	Type of study	Region	Geographic area	Simple size	Male/%	Positive/male (%)	Prevalence (%)	Score	Grade
Zhang (18)	2023	Cross-sectional	Dali (Yunnan Province)	Southern China	402	241/60%	16/NA	4.0	7	Moderate quality
Wang et al. (19)	2022	Retrospective cohort	Guigang (Guangxi Province)	Southern China	5,463	3,575/65.4%	2,010/NA	36.8	7	High quality
Lü et al. (20)	2022	Retrospective cohort	Beijing	Northern China	10,693	NA	134/94.0%	1.3	6	Moderate quality
Lü et al. (21)	2022	Retrospective cohort	Shanghai	Southern China	3,787	3,515/92.81%	586/93.3%	15.5	7	High quality
Li et al. (22)	2024	Retrospective cohort	Zhengzhou (Henan Province)	Northern China	2,981	2,672/89.6%	500/NA	16.8	8	High quality
Ji et al. (23)	2023	Retrospective cohort	Henan Province	Northern China	44,528	30,705/68.96%	13,385/NA	30.1	7	High quality
Guo et al. (24)	2022	Retrospective cohort	Hubei Province	Southern China	881	661/75.03%	304/NA	34.5	7	High quality
Chen et al. (25)	2024	Retrospective cohort	Liuan (Anhui Province)	Southern China	1,834	1,477/80.53%	444/80.9%	24.2	7	High quality
An et al. (26)	2022	Retrospective cohort	Yunnan Province	Southern China	93,944	56,346/60.0%	21,203/NA	22.6	7	High quality
Zhang (27)	2023	Retrospective cohort	Hunan Province	Southern China	22,231	16,671/74.99%	3,549/79.9%	16.0	7	High quality
Wen et al. (28)	2023	Cross-sectional	Guangzhou (Guangdong Province)	Southern China	4,826	NA	128/NA	2.7	8	High quality
Li (29)	2019	Retrospective cohort	Guangzhou (Guangdong Province)	Southern China	5,394	NA	176/63.1%	3.3	6	Moderate quality
Bai et al. (30)	2022	Retrospective cohort	Beijing	Northern China	10,124	NA	1,113/NA	11.0	8	High quality
Chen et al. (31)	2022	Retrospective cohort	Taiwan	Southern China	3,582	NA	61/NA	1.7	8	High quality
Ding et al. (32)	2022	Prospective cohort	Shenyang (Liaoning Province)	Northern China	1,288	1,211/94%	81/NA	6.3	7	High quality
Hsu et al. (33)	2022	Retrospective cohort	Taiwan	Southern China	1,078	1,034/95.9%	86/NA	8.0	8	High quality
Lao et al. (34)	2024	Retrospective cohort	Beijing	Northern China	830	757/91.2%	53/NA	6.4	7	High quality
Li et al. (35)	2021	Retrospective cohort	Beijing	Northern China	8,098	7,636/94.3%	427/NA	5.3	8	High quality
Zhang et al. (15)	2020	Retrospective cohort	Shenyang (Liaoning Province)	Northern China	2,155	2,001/92.9%	835/NA	38.7	7	High quality
Zhang et al. (36)	2024	Retrospective cohort	Hangzhou (Zhejiang)	Southern China	1,568	1,484/94.6%	174/NA	11.1	7	High quality

### Quality of included studies

Table 1 presents study quality and bias risk assessments. Findings indicated high overall quality among included studies, with 17 classified as high quality and 3 as moderate quality. The mean quality score for 18 cohort studies was 7.17. Major critical score deductions were attributed to inadequate comparability between exposed and non-exposed groups and insufficient cohort follow-up duration. Details of the quality evaluation are presented in Supplementary Table S2-S3.

### The prevalence of LLV among PLWH in China

**Figure 2:**
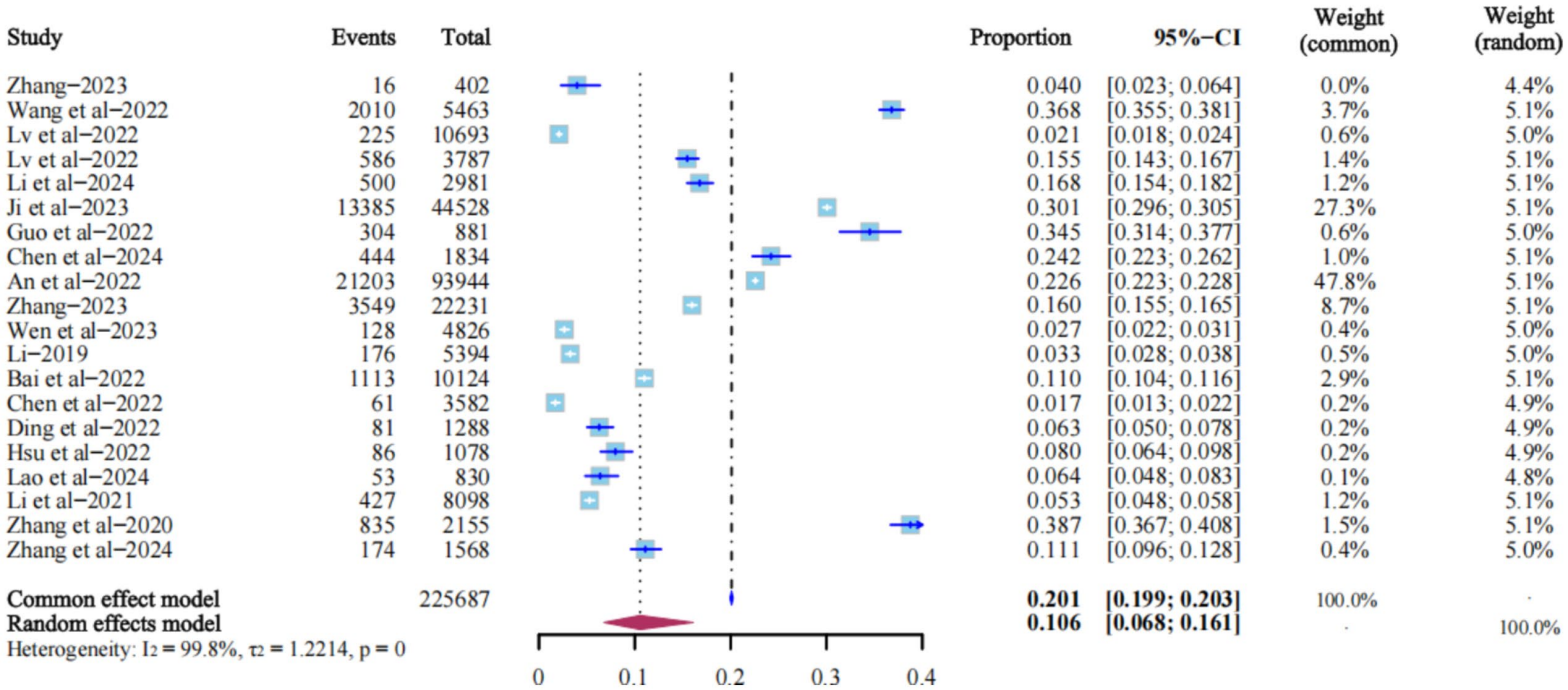
Forest plot showing the prevalence of LLV among PLWH in China.

All 20 studies reported the prevalence of LLV. The heterogeneity test *I*^2^ = 99.8%, *p* = 0, so the random effects model was used. The results showed that the prevalence of LLV among PLWH in China was 10.6% (95% CI: 6.8% ~ 16.1%) (Figure 2).

### Sensitivity analysis

**Figure 3:**
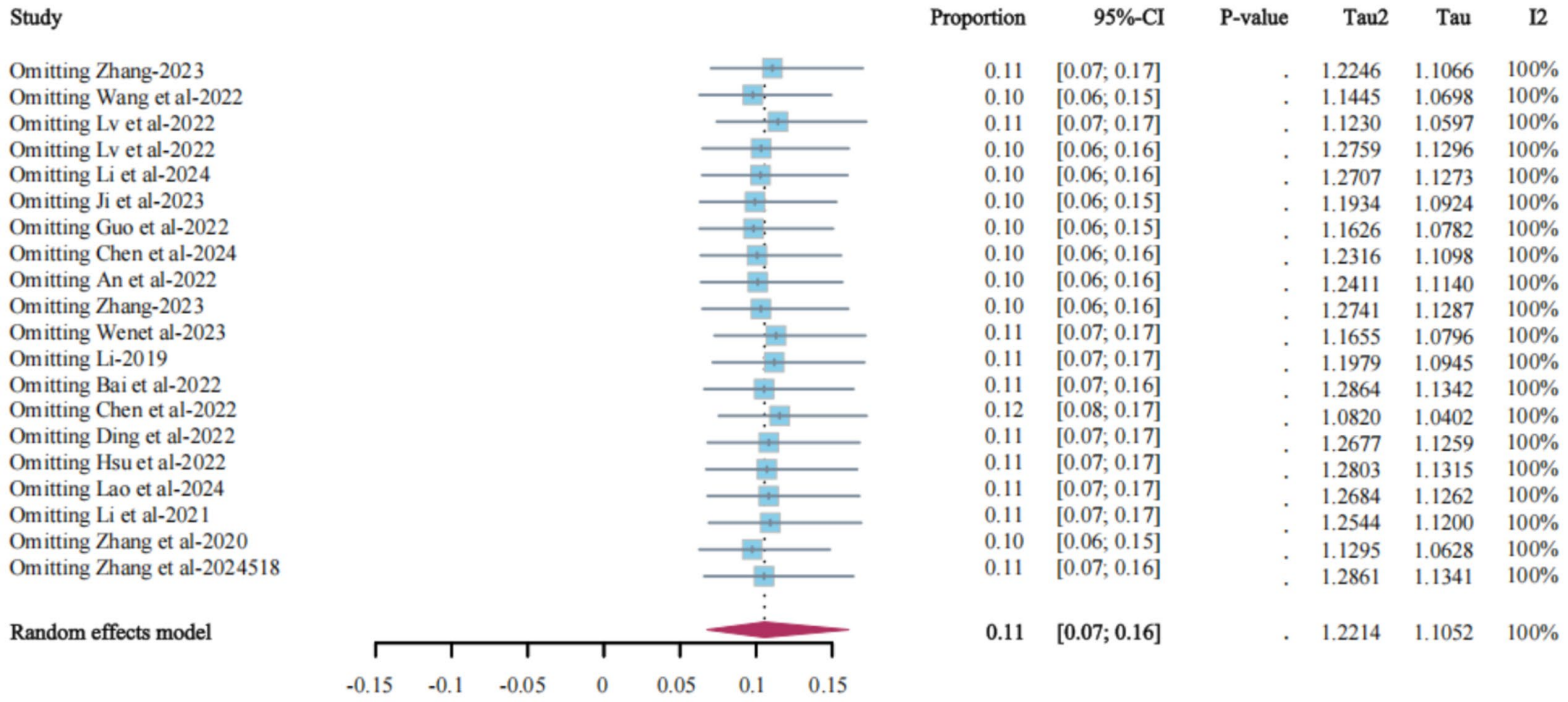
Sensitivity analysis of the meta-analysis of the prevalence of LLV among PLWH in China.

Sensitivity analysis was performed by excluding studies individually. Despite the high heterogeneity, the change in the combined effect size was relatively small after each study was excluded, indicating that the results were relatively robust and not overly influenced by any single study. The combined prevalence of LLV among PLWH ranged from 10% (95% CI: 6% ~ 15%) to 12% (95% CI: 8% ~ 17%). We concluded that the prevalence of LLV was between these two values (Figure 3).

### Subgroup analysis

To identify sources of heterogeneity, subgroup analyses were conducted for frequency of LLV, VL level, geographic regions, type of study and treatment regimens. Results showed Blip and pLLV prevalence rates of 14.2% (95% CI: 7.2% ~ 23.2%) and 2.6% (95% CI: 0.9% ~ 5.1%), cross-sectional study and cohort study prevalence rates of 3.0% (95% CI: 1.9% ~ 4.4%) and 14.0% (95% CI: 8.8% ~ 20.1%) (*p* < 0.05). VL level analysis revealed prevalence rates of 9.0%(5.5% ~ 13.3%) for 50 ~ 199 cp/mL, 3.3% (1.8% ~ 5.3%) for 200 ~ 399 cp/mL, and 3.0% (1.4% ~ 5.0%) for 400 ~ 1,000 cp/mL, with the 50 ~ 199 cp/mL range showing the highest prevalence (*p* < 0.05). Geographically, although the prevalence of LLV in southern regions was higher than that in northern regions, the difference was not statistically significant (*p* > 0.05). Among treatment regimens, second-line protease inhibitor (PI)-based regimens had the highest LLV prevalence, though differences compared to NNRTI- or INSTI-based regimens were not statistically significant (*p* > 0.05) (Table 2). Subgroup analysis forest plot was shown in Supplementary Figure S1.

**Table 2:** Subgroup analysis results.

Subgroup	Studies, *n*	Heterogeneity across the studies		Effects model	Proportion (95%CI)	*p*-value
		*I* ^2^	*p*-value			
Frequency of LLV						<0.01
Blip	6	100%	0	RE	14.2% (7.2% ~ 23.2%)	
pLLV	6	100%	0	RE	2.6% (0.9% ~ 5.1%)	
VL level						<0.01
50 ~ 199 cp/mL	13	100%	0	RE	9.0% (5.5% ~ 13.3%)	
200 ~ 399 cp/mL	11	99%	0	RE	3.3% (1.8% ~ 5.3%)	
400 ~ 1,000 cp/mL	11	100%	0	RE	3.0% (1.4% ~ 5.0%)	
Geographic area						0.93
Southern	12	100%	0	RE	12.8% (6.8% ~ 20.4%)	
Northern	8	100%	0	RE	12.4% (5.2% ~ 22.0%)	
Type of study						<0.01
Cross-sectional	2	58%	0.12	RE	3.0% (1.9% ~ 4.4%)	
Cohort study	18	100%	0	RE	14.0% (8.8% ~ 20.1%)	
Regimen at initiation						0.70
NNRTI-based regimen at initiation	12	100%	0	RE	19.0% (12.1% ~ 26.9%)	
PI-based regimen at initiation	12	98%	<0.01	RE	24.3% (13.9% ~ 36.5%)	
INSTI-based regimen at initiation	4	96%	<0.01	RE	23.3% (3.5% ~ 52.1%)	

RE, Random Effects Model.

### Publication bias analysis

**Figure 4:**
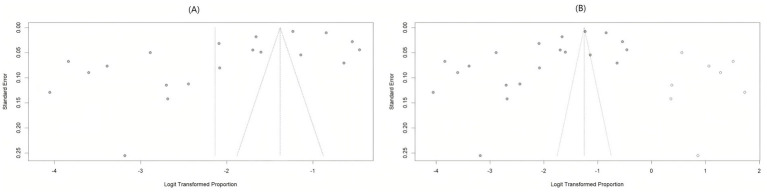
Funnel plot for risk of bias assessment. **(A)** Risk assessment funnel plot for original bias; **(B)** the funnel plot after correction by trim and fill method.

The funnel plot for bias assessment exhibited asymmetry. Egger’s test showed a significant result (*t* = −2.93, *p* = 0.0089), while Begg’s test was non-significant (*z* = −1.30, *p* = 0.1944), suggesting some publication bias in this meta-analysis. Following publication bias correction using the trim and fill method (8 missing studies imputed), the pooled prevalence of LLV increased from 10.6% (95% CI: 6.8% ~ 16.1%) to 22.2% (95% CI: 13.2% ~ 35.0%) indicating that our results might have been underestimated (Figure 4).

### Influencing factors of LLV among PLWH

There were a total of 9 influencing factors for the effect size combination. Depending on the degree of heterogeneity, the common effect model and the random effect model were selected, respectively. Our study showed that baseline CD4 < 200 cells/μL (OR = 1.398, 95% CI: 1.318 ~ 1.484), baseline CD4 200 ~ 350 cells/μL (OR = 1.290, 95% CI: 1.207 ~ 1.379), PI-based regimen at initiation (OR = 1.377, 95% CI: 1.157 ~ 1.640), change the treatment schemes (OR = 1.883, 95% CI: 1.790 ~ 1.981), baseline VL > 10^5^cp/mL (OR = 1.567, 95% CI: 1.340 ~ 1.833), poor ART adherence (OR = 3.019, 95% CI: 1.091 ~ 8.353), age ≥ 50 years (OR = 1.228, 95% CI: 1.135 ~ 1.474), and age of ART initiation ≥ 50 years (OR = 1.219, 95% CI: 1.161 ~ 1.280) were risk factors for the occurrence of LLV among PLWH. Homosexual transmission (OR = 0.523, 95% CI: 0.363 ~ 0.753) was a protective factor for LLV (*p* < 0.001) (Table 3). Analysis of influencing factors forest map was provided in Supplementary Figure S2.

**Table 3:** Results of meta-analysis of influencing factors.

Influencing factors	Studies, *n*	Heterogeneity across the studies		Effects model	Combined effect size		*p*-value
		*I* ^2^	*p*-value		OR (95%CI)	*Z*	
Baseline CD4 < 200 cells/μL	7	0.00%	0.69	CE	1.398 (1.318 ~ 1.484)	11.07	<0.0001
Baseline CD4 200 ~ 350 cells/μL	2	0.00%	0.34	CE	1.290 (1.207 ~ 1.379)	7.51	<0.0001
PI-based regimen at initiation	4	81.336%	<0.01	RE	1.377 (1.157 ~ 1.640)	3.60	0.0003
Change the treatment schemes	3	0.000%	0.99	CE	1.883 (1.790 ~ 1.981)	24.50	<0.0001
Baseline VL > 10^5^ cp/mL	3	15.488%	0.31	CE	1.567 (1.340 ~ 1.833)	5.63	<0.0001
Poor ART adherence	3	94.553%	<0.01	RE	3.019 (1.091 ~ 8.353)	2.13	0.0333
Age ≥ 50 years	2	0.000%	0.91	CE	1.293 (1.135 ~ 1.474)	3.87	<0.0001
Age of ART initiation ≥ 50 years	2	44.672%	0.18	CE	1.219 (1.161 ~ 1.280)	7.97	<0.0001
Homosexual transmission	2	39.111%	0.20	CE	0.523 (0.363 ~ 0.753)	−3.48	0.0005

CE, Common Effect Model; RE, Random Effects Model.

## Discussion

The “China AIDS Control and Prevention Plan (2024–2030)” aims to achieve the public health goal of ending AIDS by 2030 (37). While China has made substantial progress in AIDS prevention and control, persistent LLV poses risks of HIV transmission, VF, and drug resistance, challenging the realization of this goal. This systematic review synthesized data on LLV prevalence and associated factors among PLWH in China. Findings showed an overall LLV prevalence of 10.6%, with significant risk factors including baseline VL > 10^5^ cp/mL, baseline CD4 < 350 cells/μL, initiation of protease inhibitor (PI)-based regimens, treatment regimen changes, poor ART adherence, age ≥ 50 years, and ART initiation at ≥ 50 years. Homosexual transmission was identified as a protective factor.

Our study revealed an overall LLV prevalence of 10.6% among PLWH in China, which is lower than the global rate (13.81%) reported by Zhao et al., and consistent with the domestic prevalence (10.12%) documented in this article (38). However, this study was subject to some publication bias, potentially attributed to limited sample size of included studies, heterogeneity in patient demographics (e.g., baseline CD4 counts, ART regimens, follow up durations), variations in VL detection sensitivity, and diverse data sources. Following bias correction via the trim and fill method, the pooled LLV prevalence increased from 10.6 to 22.2%, suggesting the true prevalence among PLWH may be even higher. These findings indicate prior studies might have underestimated LLV risk, prompting clinicians to remain alert for virologic “pseudosuppression” while advocating for standardized LLV definitions and enhanced reporting of negative outcomes.

Subgroup analysis of LLV prevalence revealed that the incidence of Blip viraemia was significantly higher than pLLV among PLWH. Previous studies have confirmed that viral blips are closely associated with abnormal immune activation (39). For such patients, increasing the frequency of VL testing and closely monitoring intra-patient viral dynamics are recommended. Timely intervention should be implemented to prevent disease progression and exacerbation. Our analysis further revealed a higher prevalence of LLV in the 50 ~ 200 cp/mL VL range compared to 201 ~ 400 cp/mL and 401 ~ 1,000 cp/mL tiers. Zhang et al. demonstrated that LLV confers an increased risk of VF only when VL reaches ≥ 400 cp/mL (15). However, emerging evidence indicated that patients with 50 ~ 200 cp/mL VL still exhibited significantly higher risks of virological non-suppression and treatment failure compared to those with persistently undetectable VL (40, 41). The southern region exhibited a slightly higher prevalence than the northern region, indicating that the prevalence of LLV varies by region in China. Differences in regional economic levels, ART regimens, VL detection technologies, and detection frequencies all lead to variations in the prevalence rates (42). However, the impact of these factors on the prevalence rate varies among different studies, and it is necessary to analyze them specifically according to different circumstances. Kanapathipillai et al. found that high-income regions perform VL testing more frequently than middle and low-income regions (43). This allows earlier detection of LLV at lower thresholds, enabling timely treatment intervention or regimen adjustment, which may explain regional prevalence disparities. Future research priorities should include upgrading detection technologies, reducing sequencing costs, enhancing assay efficiency, adopting highly sensitive VL testing, and implementing routine VL monitoring to enable prompt LLV detection and early intervention.

Baseline CD4 level is strongly associated with LLV. CD4 + T cell counts correlate positively with immune function, such that patients with low baseline CD4 levels exhibit more severe immune damage. This enhances persistent viral replication and drug resistance development, promoting LLV emergence (44). Additionally, LLV is linked to decay of viral reservoirs established prior to ART. PLWH with extremely low baseline CD4 counts typically harbor larger HIV reservoirs with prolonged decay kinetics, thereby increasing LLV risk (45).

Our analysis showed that while LLV prevalence did not differ significantly among NNRTI, PI, and INSTI-based treatment regimens, PI-based regimens exhibited numerically higher LLV rates. Notably, PI-based regimen at initiation was identified as an independent risk factor for LLV among PLWH. This could be attributed to two plausible mechanisms: (1) patients on PI-based regimens often have poorer baseline health, or switch to second-line PI regimens due to first-line ART resistance or VF, which may predispose to LLV (46); (2) prior research suggests that PI-based ART is associated with larger peripheral blood HIV reservoir sizes compared to NNRTI regimens (47).

This study identified poor ART Adherence as the most significant predictor (OR = 3.019), consistent with findings by Zaçe et al. (48). Unfortunately, among the included studies, three primary articles refer to this influencing factor (21, 22, 28), but their definitions of “poor ART adherence” are inconsistent, precluding the extraction and integration of a unified definition. Consequently, this paper only conducts a preliminary analysis of the literature mentioning this factor, without more in-depth and precise exploration. PLWH poor compliance are strongly linked to LLV and subsequent VF, likely due to missed doses leading to subtherapeutic drug concentrations and incomplete viral suppression (49, 50). Adherence management represents a key intervention target for LLV, as it significantly improves virological suppression rates in this population (51). Strategies should include comprehensive analysis of adherence determinants, patient-centered communication to address barriers, and ART regimen optimization to enhance compliance and prevent LLV.

In contrast to findings from other studies, our research identified ART treatment regimen changes as a critical risk factor for LLV among PLWH. Wen et al. demonstrated that frequent regimen changes may reduce plasma concentrations of antiretroviral drugs to subtherapeutic levels, thereby compromising viral suppression and predisposing patients to LLV (28). It has been proposed that treatment regimen changes may be associated with poor medication adherence and suboptimal disease control—factors that confer a higher risk of LLV among PLWH (25). Inter-drug variability in ADME (absorption, distribution, metabolism, excretion) properties may lead to subtherapeutic drug levels following regimen shifts. Additionally, drug–drug interactions (DDI) between ART or with co-administered medications, drug-food interactions, and absorption issues may reduce drug concentrations, increasing the risk of LLV. Recommendations include considering pharmacological factors, with ART regimen optimization guided by resistance test results, patient adherence, regimen resistance barriers, HIV RNA levels, and other treatment history. This also should account for DDI, drug-food interactions, adverse events, pill burden, and dietary requirements (52, 53).

Notably, age ≥ 50 years and ART initiation age ≥ 50 years were identified as independent risk factors for LLV, which is consistent with previous findings. Older adults typically exhibit lower educational attainment and risk awareness, accompanied by insufficient medication adherence. Additionally, they tend to have a suboptimal immune response to treatment, ultimately contributing to adverse clinical outcomes (54). Further studies have demonstrated that in older population receiving ART, age-related impairments in protein homeostasis, dysregulated stress responses, cellular senescence, and chronic inflammation are more likely to interfere with *in vivo* drug activity, diminish therapeutic efficacy, and thereby increase susceptibility to LLV (55). Mechanistically, LLV primarily arises from HIV reservoirs harbored in immature T cells of infected individuals (56). A larger HIV reservoir size and delayed viral clearance are associated with a higher propensity for LLV induction. Consequently, patients who initiate ART at a later stage face an elevated risk of developing LLV (57). In conclusion, to reduce the incidence of LLV, early initiation of ART should be prioritized for PLWH. Moreover, increased frequency of VL monitoring is recommended for older population, enabling timely detection and management of LLV.

This study identified a baseline VL > 10^5^ cp/mL as a risk factor for LLV. VL is a key indicator for assessing the efficacy of ART, which functions by suppressing VL to undetectable levels through combination regimens—thereby delaying disease progression and extending survival. Baseline VL levels dictate the extent of immune reconstitution in AIDS patients post-ART; a high baseline VL (>10^5^ cp/mL) reflects severe immune dysfunction, rendering such patients more susceptible to LLV (58). Single-cell sequencing data reveal that 23.5% of CD4 + T cells in patients with high baseline VL harbor proviral DNA, with integration sites predominantly near oncogenes—potentiating reactivation of latent viruses, a critical mechanism underlying LLV (59, 60). Additionally, high baseline VL correlates with a larger HIV reservoir, which exhibits enhanced capacity for releasing detectable virus, thereby increasing treatment difficulty and LLV risk (61). Thus, for confirmed PLWH with high baseline VL, early initiation of ART is imperative to facilitate immune reconstitution, prevent the establishment of a large viral reservoir, and mitigate LLV risk.

Significantly, homosexual transmission was identified as a protective factor for LLV. This may be ascribed to the relatively active social organizations among MSM. These organizations facilitate earlier detection of HIV infection, timely access to ART, and consistent follow-up interventions (25). Furthermore, following ART initiation, the viral suppression rate in this population rises rapidly—an outcome that ultimately lowers their risk of developing LLV (62).

Our systematic review and meta-analysis demonstrate several strengths regarding representativeness. First, the included studies encompass 12 cities across both southern and northern China, covering both economically developed and underdeveloped regions, which largely captures the major cities with HIV-infected populations in China. Second, prior studies have demonstrated that in medical research, studies with a sample size < 200 tend to systematically overestimate effect sizes in meta-analyses (63). Given the typical sample sizes of observational and cross-sectional studies in HIV/AIDS research, studies with a sample size of less than 300 were excluded to minimize potential effect size bias and overall risk of bias. Notably, most of the included studies (*n* = 15) reported age and sex distribution data, which largely reflect the epidemiological characteristics of PLWH with LLV. Furthermore, among the 20 included studies, 18 adopted the LLV diagnostic criterion of 50 ~ 1,000 cp/mL, and two studies simultaneously employed both the 50 ~ 1,000 cp/mL and 50 ~ 200 cp/mL criteria, which enhanced the robustness of the conclusions.

However, there are also several limitations: (1) The limited number of included studies, potentially influenced by study design and inclusion/exclusion criteria, warrants future re-analysis with expanded inclusion of high quality research. (2) Most included studies did not report data on VL detection methods or testing frequencies. The absence of such information may introduce uncertainty into our results. Future studies should prioritize evaluating outcomes associated with different detection methods and frequencies, rather than relying solely on pooled analyses of heterogeneous data. (3) The included studies had different definitions for certain influencing factors (such as “poor ART adherence”), which made it impossible to conduct more precise and in-depth analyses. In the future, this issue should be given due attention, and efforts should be made to promote the standardization and unification of standards and definitions in the field of AIDS, so as to carry out clinical research of higher quality and higher standards. (4) Evidence of publication bias was detected, and despite subgroup analysis of LLV prevalence, significant heterogeneity persisted across study results. Thus, these findings should be interpreted with caution.

## Conclusion

This study demonstrates that the prevalence of LLV among PLWH is moderate to low following active ART in China. However, the results exhibit substantial heterogeneity. After adjusting for publication bias, the estimated LLV prevalence increased, indicating that the LLV burden in China has been underestimated. Given the adverse clinical consequences of LLV, screening for LLV-related risk factors prior to ART initiation is warranted. Future research should prioritize prospective studies to standardize LLV management and monitoring protocols, as well as implement early interventions tailored to identified risk factors.

## Data Availability

The original contributions presented in the study are included in the article/Supplementary material, further inquiries can be directed to the corresponding author.
