# Supplementary material for: Prevalence of low-level viremia and related influencing factors among people living with HIV in China: a systematic review and meta-analysis
Source: Front Public Health. 2025 Oct 2;13:1661253. doi: 10.3389/fpubh.2025.1661253 (PMC12528013; doi:10.3389/fpubh.2025.1661253)
Supplement: Supplementary file 1 [file Data_Sheet_1.PDF]

Supplementary Table 1 Search strategy for Chinese and English databases

databases	Search formula
CNKI	1 (Topic= "艾滋病" OR "获得性免疫缺陷综合征" OR "HIV" OR "AIDS" OR "人体免疫缺陷病毒")
	2 (Topic = "低病毒血症" OR "低病毒载量")
	3 1 AND 2
WanFang	(Topic:"艾滋病" OR "获得性免疫缺陷综合征" OR "HIV" OR "AIDS" OR "人体免疫缺陷病毒") AND (Topic:"低病毒血症" OR "低病毒载量")
VIP	(Title Keyword = 艾滋病 + 获得性免疫缺陷综合征 + HIV + AIDS + 人体免疫缺陷病毒) AND (Title Keyword = 低病毒血症 + 低病毒载量)
CBM	（"艾滋病"[Title]OR"HIV"[Title]OR"AIDS"[Title]OR"获得性免疫缺陷综合征"[Title]OR"人体免疫缺陷病毒"[Title]）AND（"低病毒血症"[Title]OR"低病毒载量"[Title])
PubMed	1 (((HIV[MeSH Terms]) OR (Acquired Immunodeficiency Syndrome[MeSH Terms])) OR (Human Immunodeficiency Viruses[Title/Abstract])) OR (AIDS[Title/Abstract])
	2 ((low level viraemia[Title/Abstract]) OR (hypoviremia[Title/Abstract])) OR (low level viremia[Title/Abstract])
	3 1 AND 2
EMBASE	1 'hiv'/exp OR 'human immunodeficiency virus'/exp OR 'aids'/exp OR 'acquired immunodeficiency syndrome'/exp
	2 'low level viraemia':ab,kw,ti OR 'hypoviremia':ab,kw,ti OR 'low level viremia':ab,kw,ti
	3 1 AND 2
Cochrane library	1 ("hiv"):ab,kw,ti OR ("human immunodeficiency virus"):ab,kw,ti OR ("aids"):ab,kw,ti OR ("acquired immunodeficiency syndrome"):ab,kw,ti
	2 ("low level viraemia"):ab,kw,ti OR ("hypoviremia"):ab,kw,ti OR ("low level viremia"):ab,kw,ti
	3 1 AND 2
Web of Science	HIV OR 'Human Immunodeficiency Viruses' OR AIDS OR 'Acquired Immunodeficiency Syndrome'(Topic) AND 'low level viremia' OR hypovolemia OR 'low level viremia'(Title)

Supplementary Table 2 Quality evaluation of the included studies (cohort study)

study	Q1	Q2	Q3	Q4	Q5	Q6	Q7	Q8	Total
Wang *et al*	1	1	1	1	1	1	1	0	7
Lv *et al*	1	1	1	1	1	1	0	0	6
Lv *et al*	1	1	1	1	1	1	1	0	7
Li *et al*	1	1	1	1	1	1	1	1	8
Ji *et al*	1	1	1	1	1	1	1	0	7
Guo *et al*	1	1	1	1	1	1	1	0	7
Chen *et al*	1	1	1	1	1	1	1	0	7
An *et al*	1	1	1	1	1	1	1	0	7
Zhang	1	1	1	1	1	1	1	0	7
Li	1	1	1	1	1	1	0	0	6
Bai *et al*	1	1	1	1	1	1	1	1	8
Chen *et al*	1	1	1	1	1	1	1	1	8
Ding *et al*	0	1	1	1	1	1	1	1	7
Hsu *et al*	1	1	1	1	1	1	1	1	8
Lao *et al*	0	1	1	1	1	1	1	1	7
Li *et al*	1	1	1	1	1	1	1	1	8
Zhang *et al*	0	1	1	1	1	1	1	1	7
Zhang *et al*	0	1	1	1	1	1	1	1	7

Q1: Representativeness of the exposed cohort: “Truly representative” or “Somewhat representative” (1 star), “Selected group” or “No description of the derivation of the cohort”(0 star)

Q2: Selection of the non-exposed cohort: “Drawn from the same community as the exposed cohort” (1 star), “Drawn from a different source” or “No description of the derivation of the non exposed cohort”(0 star)

Q3: Ascertainment of exposure: “Secure record” or “Structured interview” (1 star), “Written self report” or “No description”(0 star)

Q4: Demonstration that outcome of interest was not present at start of study: “Yes” (1 star), “No” (0 star)

Q5: Comparability of cohorts on the basis of the design or analysis controlled for confounders: “The study controls for age, sex and marital status” or “Study controls for other factors” (1 star), “Cohorts are not comparable on the basis of the design or analysis controlled for confounders”(0 star)

Q6: Assessment of outcome: “Independent blind assessment” or “Record linkage”(1 star), “Self report” or “No description” (0 star)

Q7: Was follow-up long enough for outcomes to occur: “Yes” (1 star), “No” (0 star)

Q8: Adequacy of follow-up of cohorts: “Complete follow up- all subject accounted for” or “Subjects lost to follow up unlikely to introduce bias- number lost less than or equal to 20% or description of those lost” (1 star), “Follow up rate less than 80% and no description of those lost” or “No statement” (0 star)

Supplementary Table 3 Quality evaluation of the included studies (Cross-sectional)

study	Q1	Q2	Q3	Q4	Q5	Q6	Q7	Q8	Q9	Q10	Q11	Total
Zhang	1	1	1	1	1	0	1	0	0	1	0	7
Wen *et al*	1	1	1	1	1	1	1	0	0	1	0	8

Q1: Is the source of data (survey, literature review, etc.) clearly specified?

Q2: Are the inclusion and exclusion criteria for the exposed and non-exposed groups (cases and controls) listed, or are previous publications referenced?

Q3: Is the time period for identifying patients specified (which time period of patients was included in the study)?

Q4: If the study population is not from a general population, are the study subjects consecutive (i.e., were all patients during a specific period included in the study)?

Q5: For the assessment of subjective patient indicators, are the assessors blinded to other objective indicators of the patients

Q6: Is any quality assurance assessment described

Q7: Are the reasons for excluding any patients from the analysis explained?

Q8: Is the approach to evaluating and/or controlling confounding factors described?

Q9: If there are missing values, is the method for handling missing data in the analysis explained?

Q10: Is the patient response rate and data collection completeness summarized?

Q11: If there is a follow-up, is the percentage of incomplete patient data or follow-up results provided to clarify the expected missing data?

An item was scored '1' if it received 'yes' as a response and '0' if it received 'no' or 'unclear' as a response.

A

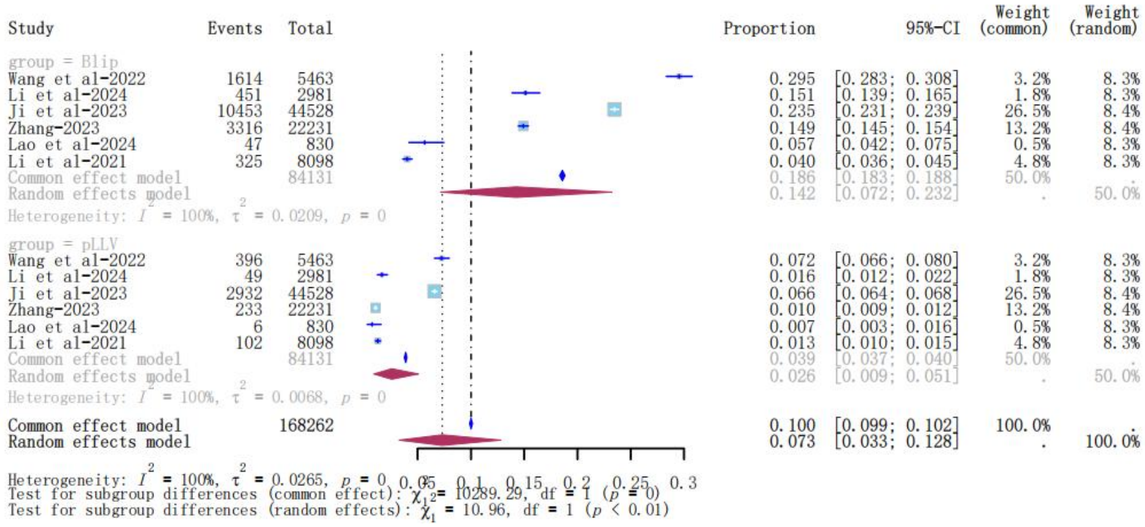

B

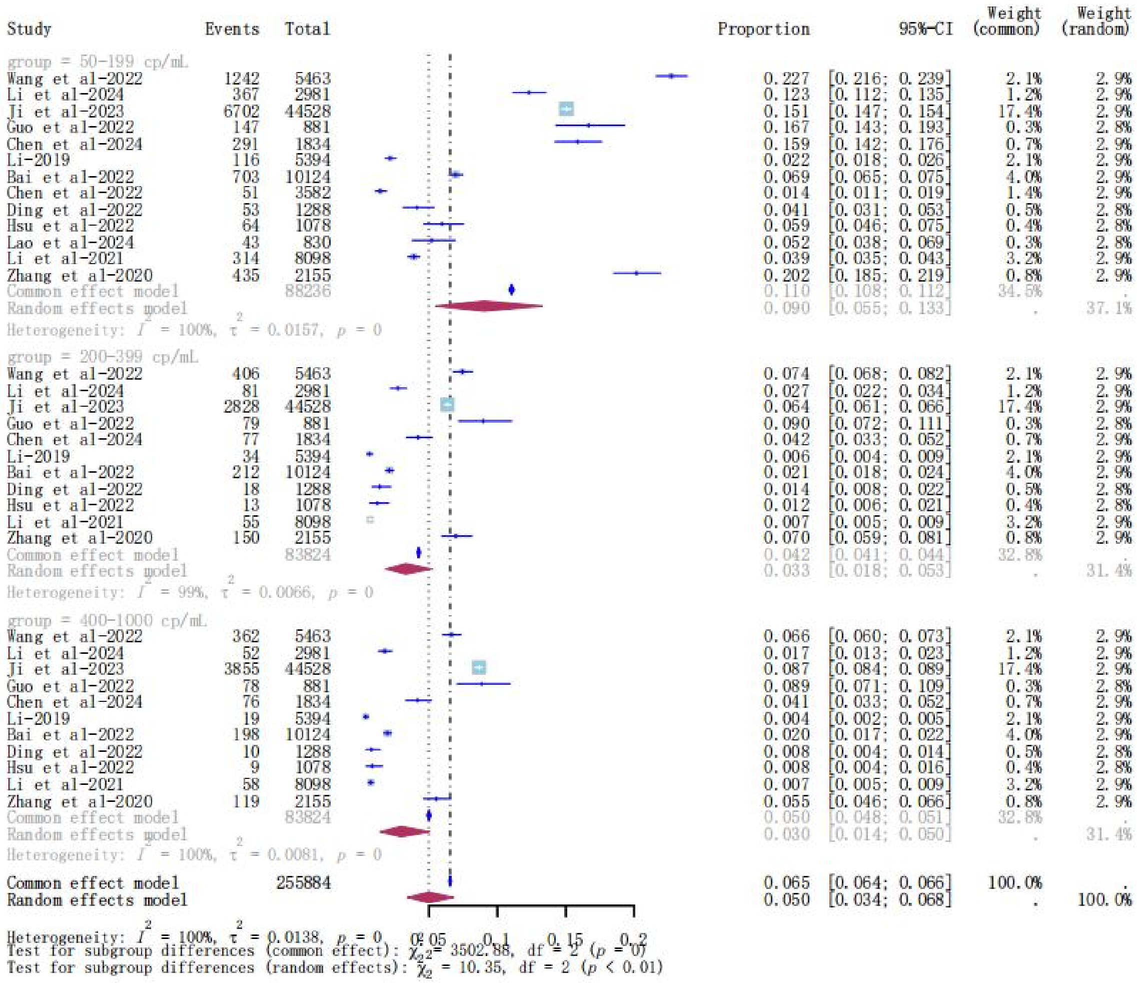
Study Events Total Proportion 95%-CI Weight (common) Weight (random)
group = 50-199 cp/mL
Wang et al-2022 1242 5463 0.227 [0.216; 0.239] 2.1% 2.9%
Li et al-2024 367 2981 0.123 [0.112; 0.135] 1.2% 2.9%
Ji et al-2023 6702 44528 0.151 [0.147; 0.154] 17.4% 2.9%
Guo et al-2022 147 881 0.167 [0.143; 0.193] 0.3% 2.8%
Chen et al-2024 291 1834 0.159 [0.142; 0.176] 0.7% 2.9%
Li-2019 116 5394 0.022 [0.018; 0.026] 2.1% 2.9%
Bai et al-2022 703 10124 0.069 [0.065; 0.075] 4.0% 2.9%
Chen et al-2022 51 3582 0.014 [0.011; 0.019] 1.4% 2.9%
Ding et al-2022 53 1288 0.041 [0.031; 0.053] 0.5% 2.8%
Hsu et al-2022 64 1078 0.059 [0.046; 0.075] 0.4% 2.8%
Lao et al-2024 43 830 0.052 [0.038; 0.069] 0.3% 2.8%
Li et al-2021 314 8098 0.039 [0.035; 0.043] 3.2% 2.9%
Zhang et al-2020 435 2155 0.202 [0.185; 0.219] 0.8% 2.9%
Common effect model 88236 0.110 [0.108; 0.112] 34.5% .
Random effects model 0.090 [0.055; 0.133] . 37.1%
Heterogeneity: I2 = 100%, τ2 = 0.0157, p = 0
group = 200-399 cp/mL
Wang et al-2022 406 5463 0.074 [0.068; 0.082] 2.1% 2.9%
Li et al-2024 81 2981 0.027 [0.022; 0.034] 1.2% 2.9%
Ji et al-2023 2828 44528 0.064 [0.061; 0.066] 17.4% 2.9%
Guo et al-2022 79 881 0.090 [0.072; 0.111] 0.3% 2.8%
Chen et al-2024 77 1834 0.042 [0.033; 0.052] 0.7% 2.9%
Li-2019 34 5394 0.006 [0.004; 0.009] 2.1% 2.9%
Bai et al-2022 212 10124 0.021 [0.018; 0.024] 4.0% 2.9%
Ding et al-2022 18 1288 0.014 [0.008; 0.022] 0.5% 2.8%
Hsu et al-2022 13 1078 0.012 [0.006; 0.021] 0.4% 2.8%
Li et al-2021 55 8098 0.007 [0.005; 0.009] 3.2% 2.9%
Zhang et al-2020 150 2155 0.070 [0.059; 0.081] 0.8% 2.9%
Common effect model 83824 0.042 [0.041; 0.044] 32.8% .
Random effects model 0.033 [0.018; 0.053] . 31.4%
Heterogeneity: I2 = 99%, τ2 = 0.0066, p = 0
group = 400-1000 cp/mL
Wang et al-2022 362 5463 0.066 [0.060; 0.073] 2.1% 2.9%
Li et al-2024 52 2981 0.017 [0.013; 0.023] 1.2% 2.9%
Ji et al-2023 3855 44528 0.087 [0.084; 0.089] 17.4% 2.9%
Guo et al-2022 78 881 0.089 [0.071; 0.109] 0.3% 2.8%
Chen et al-2024 76 1834 0.041 [0.033; 0.052] 0.7% 2.9%
Li-2019 19 5394 0.004 [0.002; 0.005] 2.1% 2.9%
Bai et al-2022 198 10124 0.020 [0.017; 0.022] 4.0% 2.9%
Ding et al-2022 10 1288 0.008 [0.004; 0.014] 0.5% 2.8%
Hsu et al-2022 9 1078 0.008 [0.004; 0.016] 0.4% 2.8%
Li et al-2021 58 8098 0.007 [0.005; 0.009] 3.2% 2.9%
Zhang et al-2020 119 2155 0.055 [0.046; 0.066] 0.8% 2.9%
Common effect model 83824 0.050 [0.048; 0.051] 32.8% .
Random effects model 0.030 [0.014; 0.050] . 31.4%
Heterogeneity: I2 = 100%, τ2 = 0.0081, p = 0
Common effect model 255884 0.065 [0.064; 0.066] 100.0% .
Random effects model 0.050 [0.034; 0.068] . 100.0%
0.05 0.1 0.15 0.2
Heterogeneity: I2 = 100%, τ2 = 0.0138, p = 0
Test for subgroup differences (common effect): χ22 = 3502.88, df = 2 (p = 0)
Test for subgroup differences (random effects): χ2 = 10.35, df = 2 (p < 0.01)

C

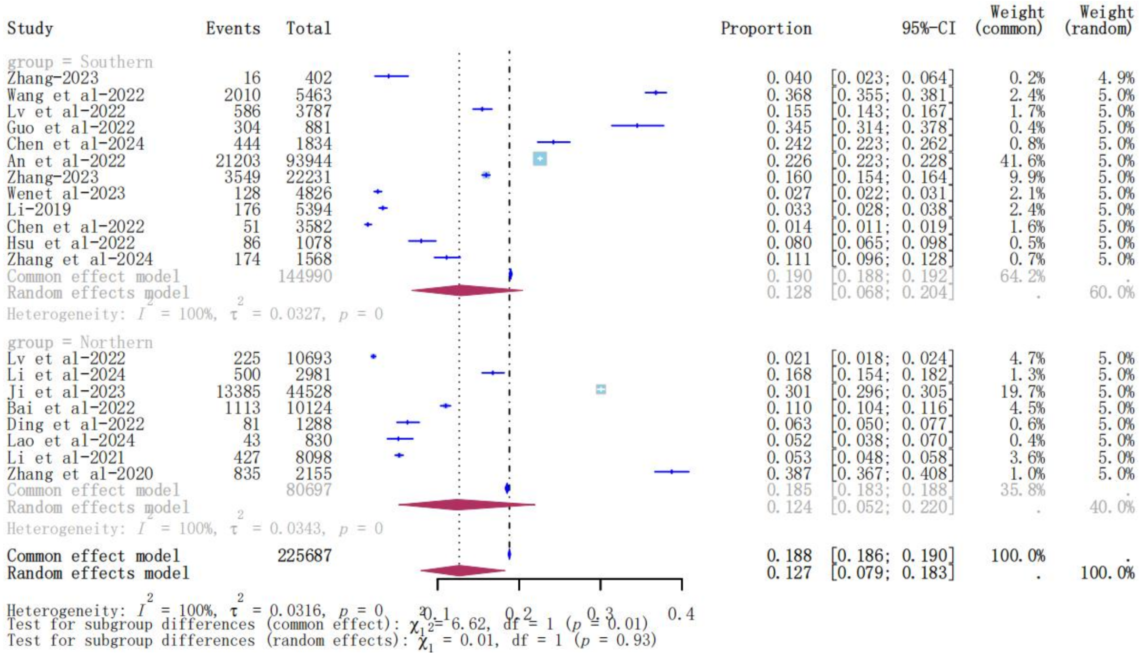
Study Events Total Proportion 95%-CI Weight (common) Weight (random)
group = Southern
Zhang-2023 16 402 0.040 [0.023; 0.064] 0.2% 4.9%
Wang et al-2022 2010 5463 0.368 [0.355; 0.381] 2.4% 5.0%
Lv et al-2022 586 3787 0.155 [0.143; 0.167] 1.7% 5.0%
Guo et al-2022 304 881 0.345 [0.314; 0.378] 0.4% 5.0%
Chen et al-2024 444 1834 0.242 [0.223; 0.262] 0.8% 5.0%
An et al-2022 21203 93944 0.226 [0.223; 0.228] 41.6% 5.0%
Zhang-2023 3549 22231 0.160 [0.154; 0.164] 9.9% 5.0%
Wenet al-2023 128 4826 0.027 [0.022; 0.031] 2.1% 5.0%
Li-2019 176 5394 0.033 [0.028; 0.038] 2.4% 5.0%
Chen et al-2022 51 3582 0.014 [0.011; 0.019] 1.6% 5.0%
Hsu et al-2022 86 1078 0.080 [0.065; 0.098] 0.5% 5.0%
Zhang et al-2024 174 1568 0.111 [0.096; 0.128] 0.7% 5.0%
Common effect model 144990 0.190 [0.188; 0.192] 64.2% .
Random effects model 0.128 [0.068; 0.204] . 60.0%
Heterogeneity: I2 = 100%, τ2 = 0.0327, p = 0
group = Northern
Lv et al-2022 225 10693 0.021 [0.018; 0.024] 4.7% 5.0%
Li et al-2024 500 2981 0.168 [0.154; 0.182] 1.3% 5.0%
Ji et al-2023 13385 44528 0.301 [0.296; 0.305] 19.7% 5.0%
Bai et al-2022 1113 10124 0.110 [0.104; 0.116] 4.5% 5.0%
Ding et al-2022 81 1288 0.063 [0.050; 0.077] 0.6% 5.0%
Lao et al-2024 43 830 0.052 [0.038; 0.070] 0.4% 5.0%
Li et al-2021 427 8098 0.053 [0.048; 0.058] 3.6% 5.0%
Zhang et al-2020 835 2155 0.387 [0.367; 0.408] 1.0% 5.0%
Common effect model 80697 0.185 [0.183; 0.188] 35.8% .
Random effects model 0.124 [0.052; 0.220] . 40.0%
Heterogeneity: I2 = 100%, τ2 = 0.0343, p = 0
Common effect model 225687 0.188 [0.186; 0.190] 100.0% .
Random effects model 0.127 [0.079; 0.183] . 100.0%
0.1 0.2 0.3 0.4
Heterogeneity: I2 = 100%, τ2 = 0.0316, p = 0
Test for subgroup differences (common effect): χ12 = 6.62, df = 1 (p = 0.01)
Test for subgroup differences (random effects): χ1 = 0.01, df = 1 (p = 0.93)

D

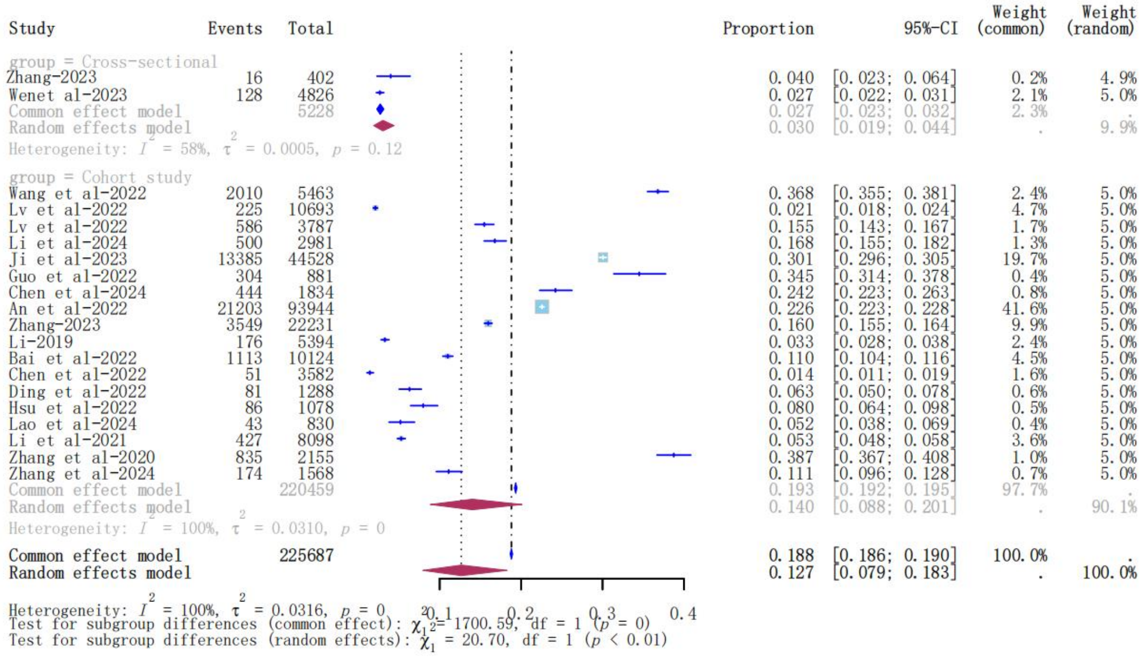

E

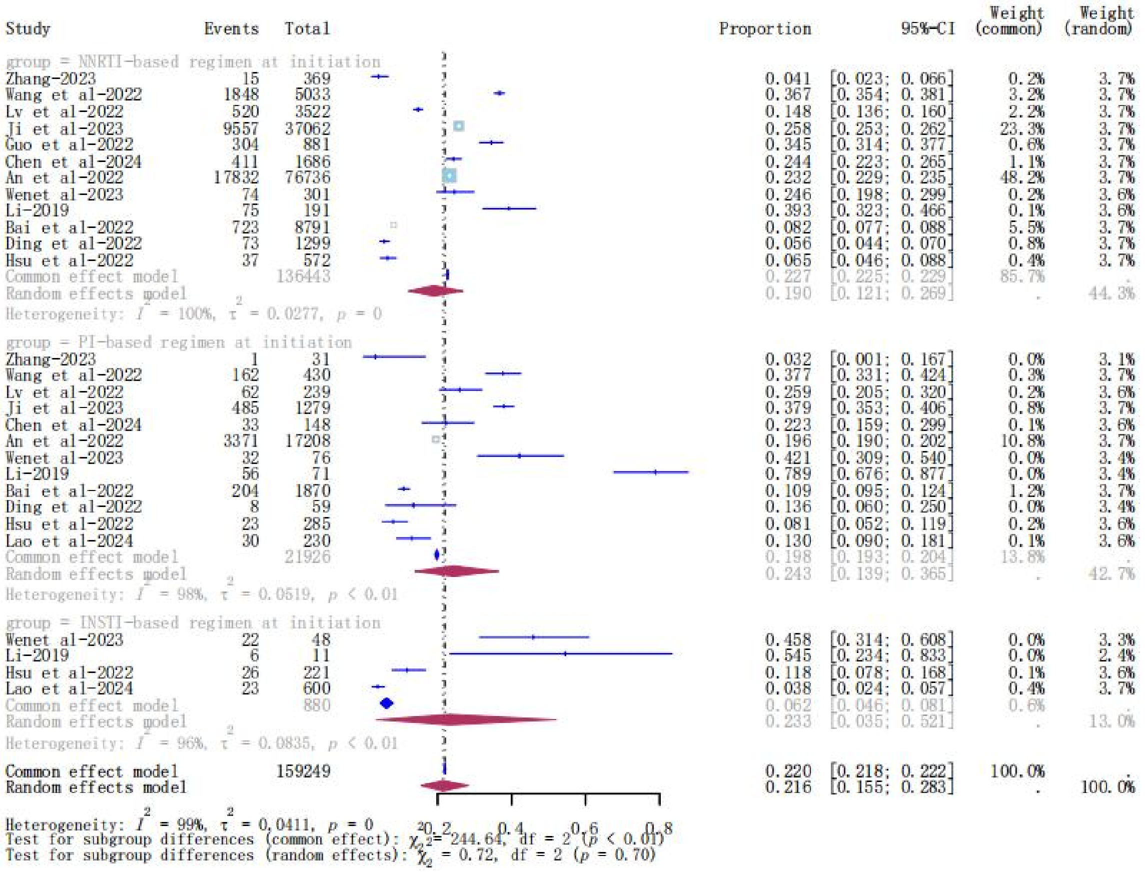

Supplementary Fig. 1 Subgroup analysis of the prevalence of LLV in AIDS patients. A: Pooled Prevalence of LLV by Frequency of LLV; B: Pooled Prevalence of LLV by VL level; C: Pooled Prevalence of LLV by geographic area; D: Pooled Prevalence of LLV by type of study; E: Pooled

Prevalence of LLV by Regimen at Initiation

A

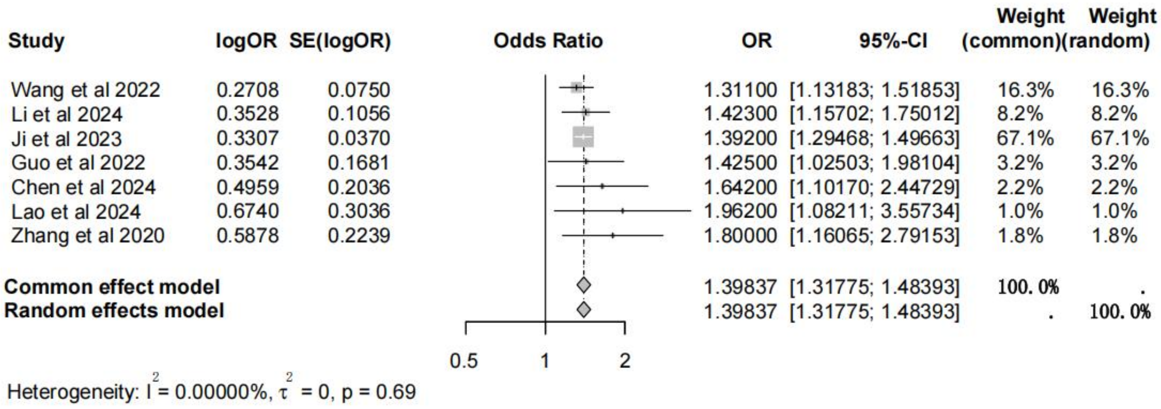

B

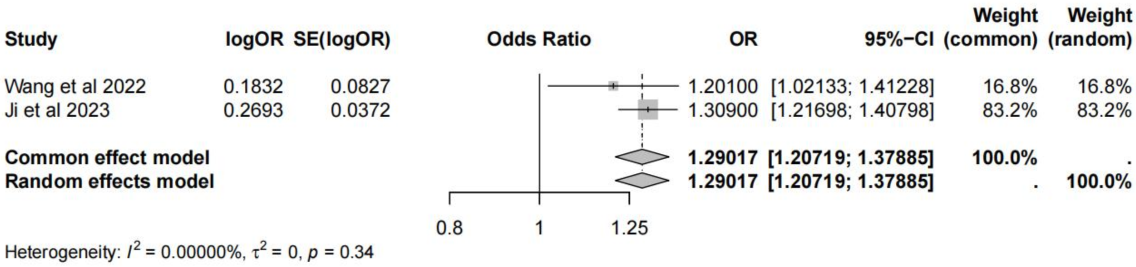

C

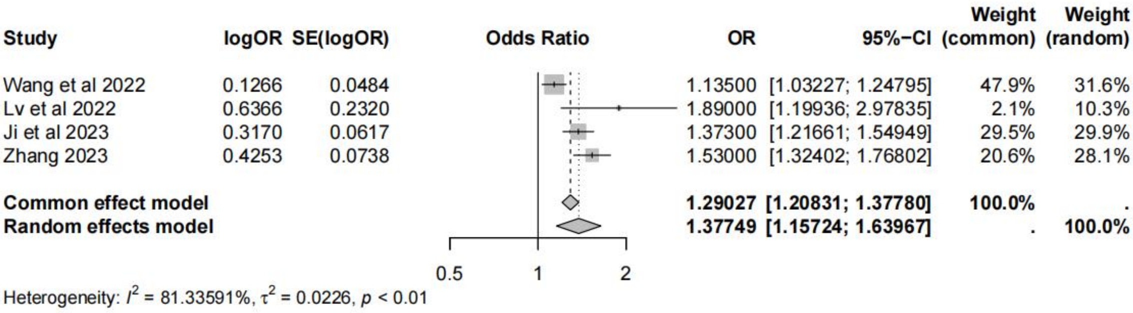

D

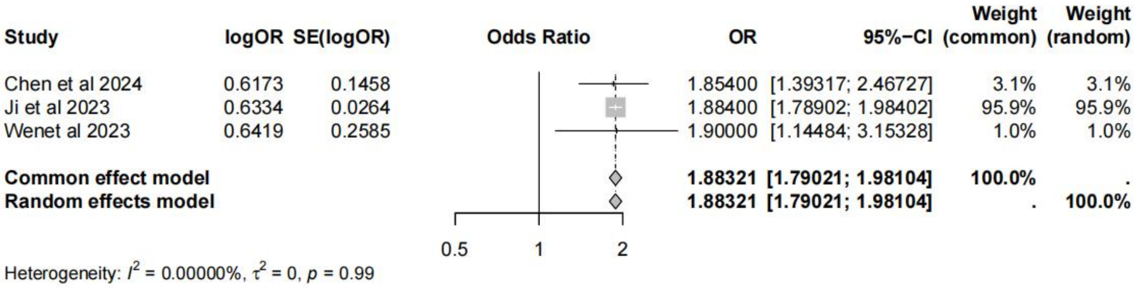

E

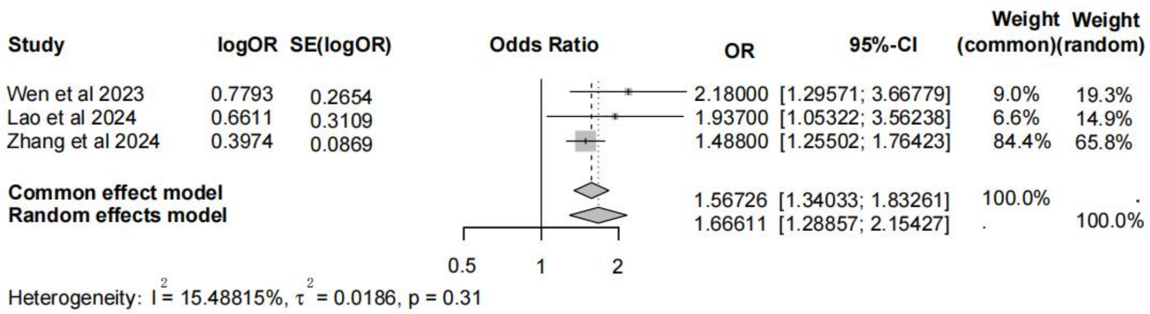

F

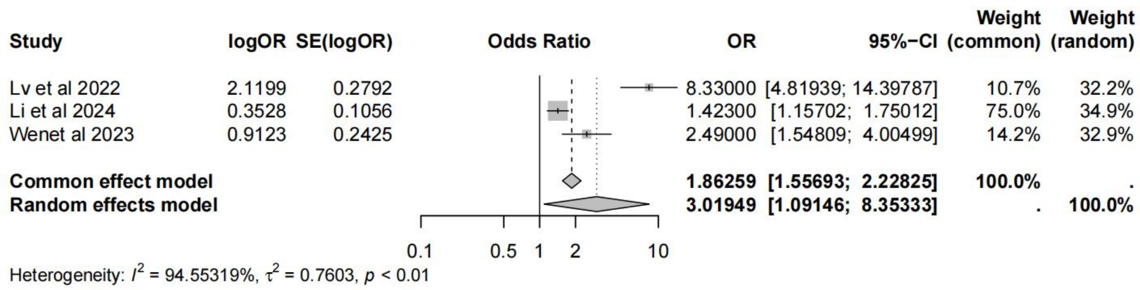

G

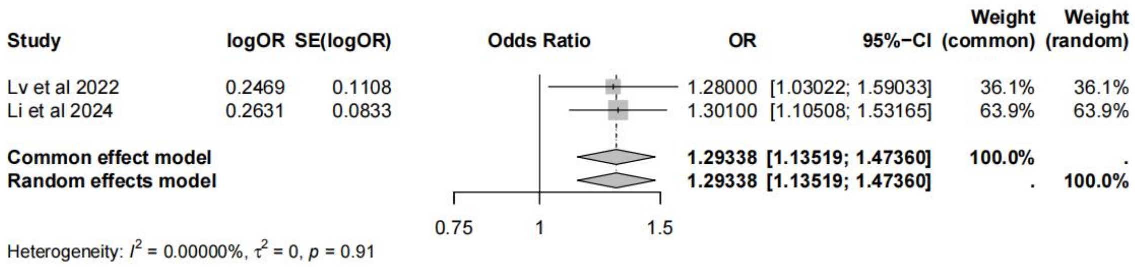

H

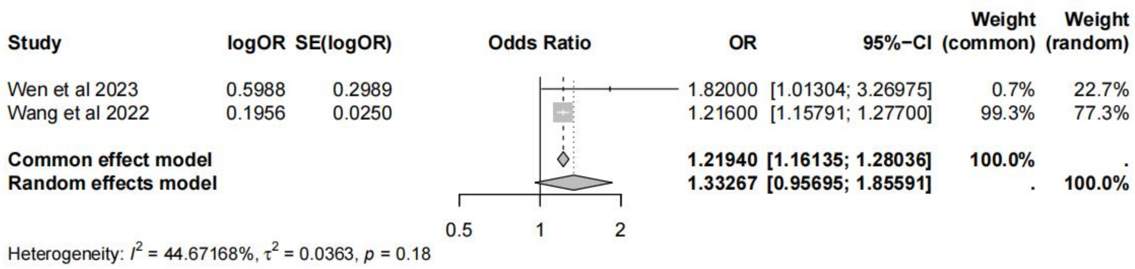

I

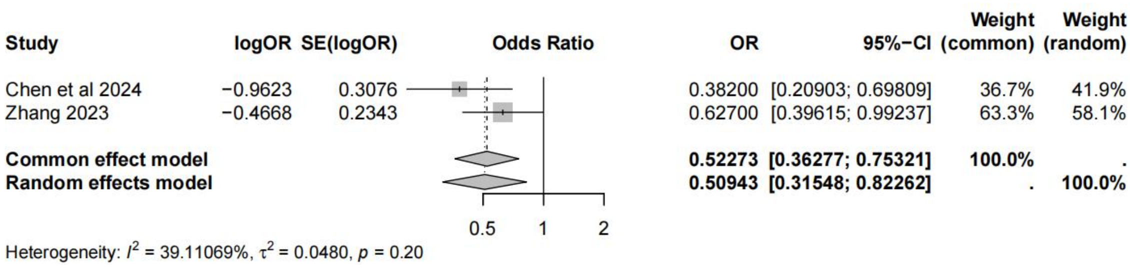

Supplementary Fig. 2 Analysis of the Influencing Factors of LLV among AIDS Patients. A: Baseline CD4＜200 cells/μL; B: Baseline CD4 200~350 cells/μL; C: PI-based regimen at initiation; D: Change the treatment schemes; E: Baseline VL＞10^5 cp/mL; F: Poor ART adherence;

G: Age≥50 years; H: Age of ART initiation≥50 years; I: Homosexual Transmission.
